# *Streptomyces* sp. 1S1 isolated from Southern coast of the Red Sea as a renewable natural resource of several bioactive compounds

**DOI:** 10.1016/j.jsps.2021.12.012

**Published:** 2021-12-31

**Authors:** Azal A. Mothana, Hassan A. Al-Shamahy, Ramzi A. Mothana, Jamal M. Khaled, Adnan J. Al-Rehaily, Abdullah Y. Al-Mahdi, Ulrike Lindequist

**Affiliations:** a Department of Biology, Faculty of Marine Sciences and Environment, Al-Hodeida University, Al-Hodeida, Yemen; b Department of Medicinal Microbiology, Faculty of Medicine and Health Sciences, Sana’a University, Sana'a, Yemen; c Department of Pharmacognosy, College of Pharmacy, King Saud University, Riyadh 11451, Saudi Arabia; d Department of Botany and Microbiology, College of Science, King Saud University, Riyadh 11451, Saudi Arabia; e Department of Microbiology, Faculty of Medicine, Lincoln University College, Malaysia; f Department of Pharmaceutical Biology, Institute of Pharmacy, University of Greifswald, Greifswald, Germany

**Keywords:** Red Sea, Bioactive compounds, Actinomycetes, *Streptomyces*, Fatty acids, Western coast of Yemen

## Abstract

Red Sea represents one of the most remarkable marine ecosystems. However, it is also one of the world's least explored areas of marine biodiversity. The aims of this investigation were therefore, to isolate marine microorganisms from the seashore sediments and water in shallow region from west Yemen coast, to assess their antimicrobial potential, to identify the highly active isolate, and to purify and identify the bioactive compounds from it. In this regard, twenty-five bacterial strains have been isolated from twenty samples and tested for their antimicrobial ability against some pathogenic bacteria and yeast by using the agar disk diffusion and agar well diffusion assay. Out of the total 25 marine actinomycetes isolates only 13 exhibited interesting antimicrobial activity. The morphological, biochemical, and phylogenetic characteristics of the potential isolate 1S1 were compatible with their classification in the genus *Streptomyces*. The 16S rRNA gene sequences have shown that the isolate 1S1 clustered with *Streptomyces longisporoflavus*. The strain *Streptomyces* sp. 1S1 was cultivated and extracted with ethyl acetate. The GC–MS study of the extract indicated the presence of certain fatty acyl compounds e.g., tetradecanoic acid, 9-octadecenoic acid, hexadecanoic acid, and 9,12,15-octadecatrienoic acid. Using chromatographic techniques, three compounds were isolated and by spectroscopic methods e.g., IR, MS and NMR structurally elucidated. The three compounds were identified as a triacylglyceride, 9-octadecenoic acid, and hexadecanoic acid. The study reinforces the evidence of the potential of *Streptomyces sp* and the ability to produce several antimicrobial compounds.

## 1 Introduction

Bacteria and other microorganisms are ubiquitous in the marine environment. They are taxonomically diverse, biologically active, and colonize all aquatic living habitats, from the deep waters to the shallowest estuaries (Rheinheimer 1992).

Bacteria, actinomycetes, and fungi among marine microorganisms in general, are considered important sources of many naturally occurring biomolecules and therapeutic products (Bredholt et al., 2008, Liu et al., 2008; and Thomas et al., 2010). Due to the cold, lightless and pressure conditions they live in a stressful habitat that leads to unique metabolisms that allow them to produce metabolites that differ from terrestrial metabolites (Rahman et al., 2010), and to adapt to extreme habitats and withstand utmost conditions such as temperature, oxygen level, high pH, pressure, nutrient limitation, salinity and osmolality (Wright et al., 2003).

In the quest for industrial essential molecules, marine microorganisms and their secondary metabolites are becoming more and more valuable. Currently, both the academic and the industrial concerns in marine microorganisms are increasing because they have recorded novel and pharmacologically active compounds (Ramesh and Mathivanan, 2009, Khajure., P.V., Rathod, J.L. , 2011**).** Moreover, it is reported that marine sediment sources are valuable for the isolating novel actinomycetes capable of producing beneficial novel products (Goodfellow and Hynes, 1984, Al-Zereini et al., 2010).

The Red Sea is one of the world's most under-studied marine biodiversity areas, but the high degree of endemism shows that other evolutionary divisions await discovery. Due to many factors, such as high evaporation levels, low precipitation, the lack of substantial rivers and the restricted relation to the Indian Ocean which caused high salinity and therefore, rendering the Red Sea one of the most important marine ecosystems (El Samak et al., 2018, Yui, 2012).

Few investigations on isolated marine actinomycetes from the Red Sea and their bioactivities have been published. In 2005 Bérdy isolated marine actinomycetes with significant antimicrobial activities from the Red Sea. Moreover, Abdelmohsen et al. (2014) isolated an actinomycetes from the Red Sea, which has shown antibacterial and antiviral properties. While Nadeem et al. (2015) identified aminoglycosides with antibacterial activity from marine actinomycetes, Sagar et al., 2013a, Sagar et al., 2013b documented the isolation of several halophilic bacterial strains from the Red Sea (Jeddah) which showed significant cytotoxic activities against various lines of cancer cells.

In several northern coastal locations of the Red Sea, an extensive study was undertaken on the isolation and cultivation of marine microorganisms. However, the coast of the Southern part and especially, Yemen's coast is relatively unexplored so far. Therefore, we have been encouraged to undertake this work. In this study, various actinomycetes and bacterial strains were isolated from different locations of the western coastal plain of Yemen, characterized and investigated for their antimicrobial potential. Moreover, the most active strain has been identified, cultivated, extracted, and antimicrobial active compounds have been isolated.

## 2 Materials and methods

### 2.1 Study area

**Fig. 1:**
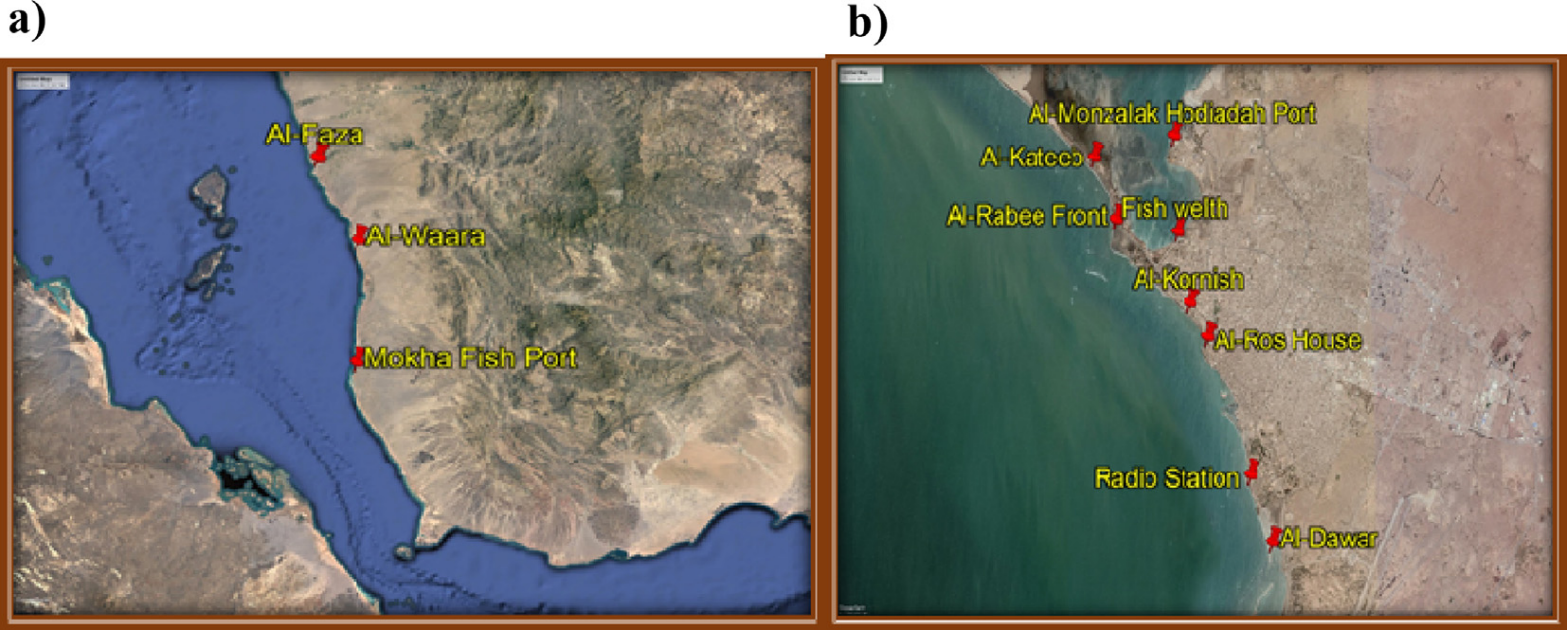
Study area, a) Location of the collected samples in Al-Hodeidah area, b) Location of collecting samples in Mokha area.

The samples were collected from 10 different sites along Tehama western coast of Yemen from Al-Hodeidah north to Al-Mokha south, lies between longitude range 42⁰ 56′ 0″0.851509 E- 43⁰ 14′ 31″.040131E and latitude range 14⁰ 49′ 52″0.9093 N – 13⁰ 19′ 18″0.475820 N, Fig. 1**a and b**.

### 2.2 Collection of marine samples

Twenty samples (10 sediment samples and 10 seawater samples) were collected from ten different sites along the west coast of Yemen in 2013–2014 and 2016–2017. Marine samples were taken from the Red Sea coast between 30 cm and 1000 cm deep. The temperature, pH and salinity of each sample were measured with thermometer, pH and salinity meter. The samples were stored at 4 °C using sterile polythene containers (Data are not shown).

### 2.3 Isolation of marine actinomycetes

Until serial dilution, both marine sediment and surface water samples were pre-heat treated. Pre-heat treatment was carried out at 50 °C with incubating water and sedimentary samples for 10 min (Saisivam et al., 2008, Al-Awadhi, 2016). The isolation and purification were done using starch casein agar (SCA) medium (HiMedia, India) prepared according to manufacturer's directions and supplemented with amphotericin B and chloramphenicol (30 µg/ml of each) (Ramesh and Mathivanan, 2009, Mohseni et al., 2013). The plates were aerobically incubated at 26 ± 3 °C for 10 days at a shaking incubator at 150 RPM. On SCA plates, daily observation showed the development and appearance of actinomycetes; they were easily detected by their distinctive calcareous to leathery appearance. Spore appearance and growth were also checked using a light microscope to see if they were filamentous. The SCA was used to individually harvest colonies and subculture them. It has been discovered that a number of characteristics are unique to the colonies, including variations in the shape, color, and level of diffusible pigment. For 5–14 days, samples were incubated at 28° C in SCA slants to generate the best possible sporulation. Marine actinomycetes isolates have been stored on (SCA I**)** at 4 °C and finally preserved at −20 °C in 20% sterile glycerol in distilled water (v/v) (Williams et al., 1971, Al-Mahdi, 2005, Al-Mekhlafi, 2007).

### 2.4 Macroscopic and microscopic features

Culture characteristics of pure actinomycetes colonies such as elevation, surface, aerial and substrate mycelium color and pigments production were recorded on SCA media according to Bergey’s Manual of Determinative Bacteriology (Holt et al., 1994). To study the isolates' microscopic characteristics, a cover slip culture method (Mitchell and Britt 1981) was performed and yielded results such as the substrate and mycelia of individual isolates, the coil and chain-shaped spores produced, forming of rectiflexibiles, unbranched and branched chains, retinaculum-apertum and spiral spores.

### 2.5 Determination of the antimicrobial activity

#### 2.5.1 Microbial organisms

Tests microorganisms as bacteria were *Bacillus subtilis* ATCC 6633, *Staphylococcus aureus* ATCC 9538, *Escherichia coli* MTCC 739, *Pseudomonas aeruginosa* MTCC 2453 and locally *Streptococcus pneumoniae* and as fungus *Candida albicans* ATCC 2091. The antimicrobial activity of the marine isolates was tested using two methods namely, agar disc diffusion method (ADM) (cup method) and agar spot inoculation. Marine actinomycetes isolates were streaked on (SCA) and incubated at 28 °C for 7 days, target microorganisms were seeded on nutrient agar (NA) for bacteria and Sabouraud dextrose agar (SDA) for fungi.

#### 2.5.2 Agar disc diffusion method (ADM)

Discs of (SCA) with growth of marine isolates were cut by cork borer (6 mm) and transferred to the surface of seeded target of microorganism's plates under aseptic conditions. These plates were kept for 1–2 h in a refrigerator to facilitate diffusion and then incubated at 37 °C for bacteria and at 28 °C for fungi. Antimicrobial activity was recorded in term of inhibition zones against target microorganism’s growth around the agar disc of marine isolates (Gurung et al., 2009, Attimarad et al., 2012).

### 2.6 Fermentation and extraction of the actinomycete isolate (1S1)

The actinomycete isolate (1S1) which showed the greatest antimicrobial activity, was tested for its extracellular antibiotic production capabilities under submerged fermentation conditions. The selected active actinomycete was inoculated into starch casein broth and incubated at 28 °C using a shaking incubator (200–250 rpm) for seven days. After incubation, the broth was filtered through Whatmannn No.1 filter paper, then the biomass of actinomycetes was removed by centrifugation at 3000 xg for 10 min. The supernatant was collected, concentrated, and extracted with equal volume of the ethyl acetate. The ethyl acetate/broth mixture was shaken for several hours in proper flasks. Using a separating funnel, the ethyl acetate layer was then removed from the water-based broth. The extraction process was repeated three times until colorlessness of the ethyl acetate extract. The collected ethyl acetate extract was dried over anhydrous sodium sulfate, filtered, and evaporated using rotary evaporator (Buechi, Switzerland). To preserve the extract, it was kept in sealed glass vials at 4 °C in a refrigerator (Al-Mekhlafi, 2007). The extract was tested for its antimicrobial property against human pathogens by the agar well diffusion assay as previously described.

### 2.7 Identification using 16S rDNA sequencing

**Fig. 2:**
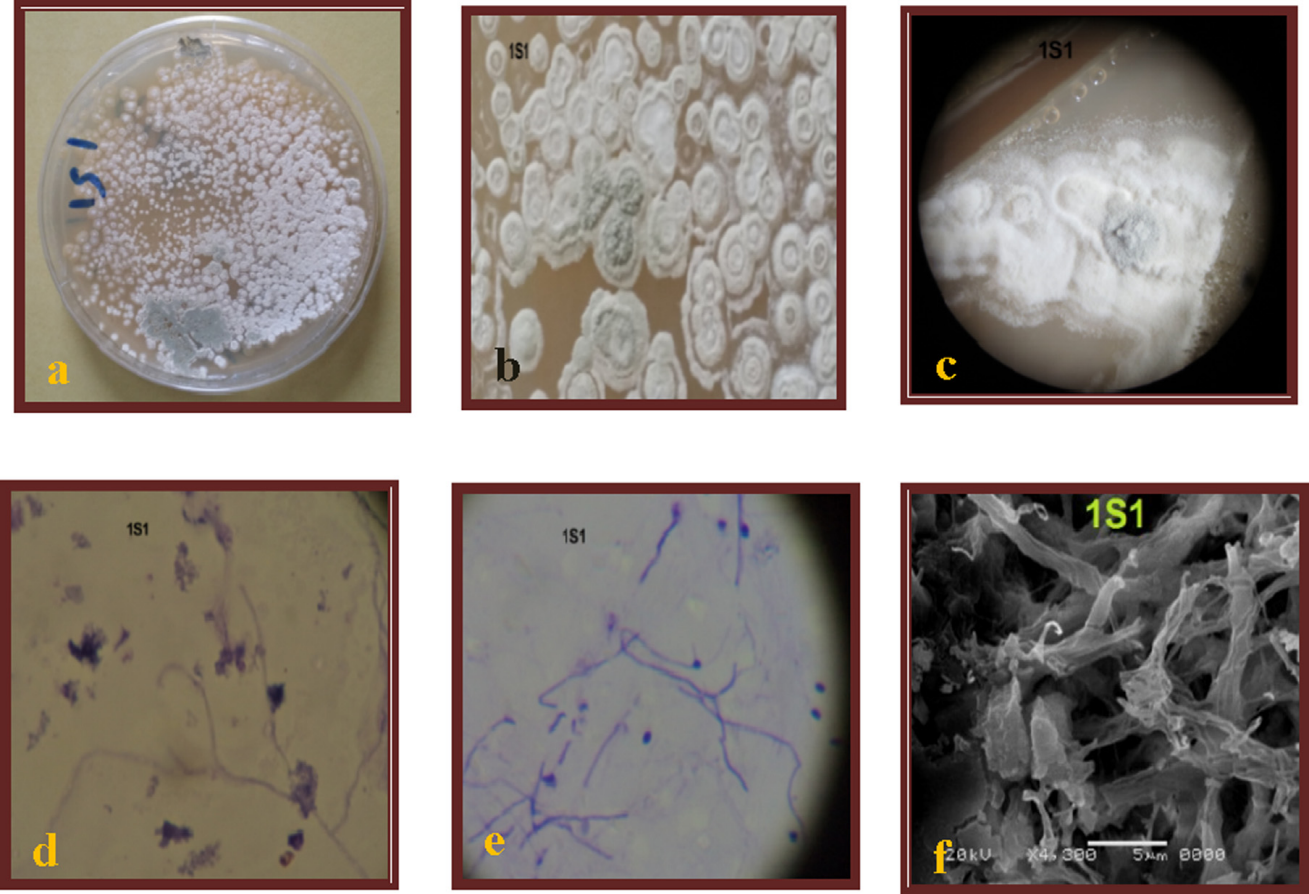
*Streptomyces longisporoflavus* (1S1). a: growth on SCA plat, b: shape of colonies, c: shape of colonies under binocular microscope, d and e: spores chain shape by cover slip, f: spores chain shape under electronic microscope.

The amplification of partial 16S rRNA gene was performed to identify the marine actinomycetes isolate (1S1) that showed the greatest antimicrobial activity against the selected human pathogens. For genetic characterization of the active isolates, the following steps were performed: Genomic DNA was extracted from the pure selected isolate (Sambrook et al., 1989). DNA preparations were then analyzed by electrophoresis according to the method described by (Sambrook et al., 1989). For DNA sequencing, the 16S rRNA gene was amplified by polymerase chain reaction (PCR) using the primer pair 8F:5′-AGAGTTTGATCCTGGCTCAG-3′1525R:5′- AAGGAGGTGWTCCARCC-3′. The PCR reaction and sequencing of the PCR product was made by the sequencing facility offered by the U.S.B. American Company through SIGMA-Egypt. The sequences of the selected isolates were analyzed using the BLAST program (www.blast.ncbi.nlm.nih.gov). The bacterial isolate was identified as *Streptomyces longisporoflavus* (Fig. 2).

### 2.8 Isolation and characterization of active principles from the ethyl acetate extract

**Scheme 1:**
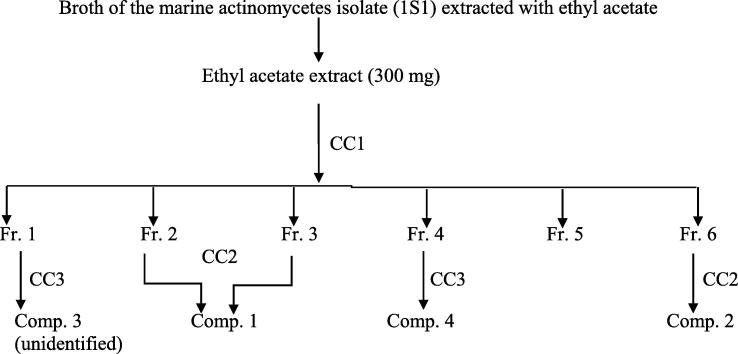
Isolation scheme of active compounds from the ethyl acetate extract.

The ethyl acetate extract was further subjected to other chromatographic separation techniques e.g., thin layer chromatography (TLC) and open column chromatography (CC) in order to isolate the main constituents. Ethyl acetate extract was chromatographed on a silica gel column (CC1, 20 mm i.d. × 400 mm) using chloroform, ethyl acetate gradient elution (9.5:0.5) until (0:100). The collected fractions (20 ml each) were pooled together depending on their TLC behavior to give six main fractions (Scheme 1).

Fractions **2** and **3** (35 mg) were rechromatographed on a reversed-phase column (CC2, 10 mm i.d. × 400 mm) (RP-18, Merck, Darmstadt, Germany) using methanol:water (5:95) as a mobile phase to afford a major substance (**compound 1**) (5 mg). On the other hand, under the same chromatographic conditions (CC2), fraction **6** (13 mg), gave **compound 2** (3 mg). While fraction **1** (15 mg) and **4** (20 mg) were separated on two reversed-phase columns (CC3, 10 mm i.d. × 400 mm) (RP-18, Merck, Darmstadt, Germany) using methanol:water (15:85) as a mobile phase to give two major substances (**compound 3**) (3 mg) and **compound 4** (3.5 mg) (Scheme 1). According to the obtained spectroscopic data, only three compounds could be characterized and identified namely, compounds 1, 2, and 4.

### 2.9 Gas chromatography-Mass spectroscopy analysis

The GC–MS examinations have been directed on gas chromatograph (Hewlett-Packard 5890 series II) connected to mass spectrometer (VG Analytical 70-250S). Fused silica packed in capillary Elite-5MS column (30 m × 0.25 mm i.d., film thickness 0.25 μm, from Perkin Elmer, USA) has been used as stationary phase. The mobile phase utilized was helium at a stream rate of 1 ml/min. Temperature of the injector was set at 200 °C. The Analysis began with heating up the oven at 60 °C and then steadily increasing it to 300 °C. The final step of the process was to keep the oven at 300 °C for a 20-minute period. An election impact ionization system with an ionization energy of 70 eV has been utilized for GC–MS detection. A scan rate of 0.6 s was utilized to cover the mass range of 35 to 600 *m*/*z*.

### 2.10 Nuclear magnetic resonance spectroscopic analysis (NMR)

The ^1^H, ^13^C and 2D-NMR spectra were recorded on a Bruker AMX-500 spectrometer. The chemical shift values are expressed in (ppm) units using tetramethylsilane (TMS) as internal standard and the coupling constants (*J*) are expressed in hertz (Hz). Spectroscopic grade of CDCl_3_ was used as a solvent.

## 3 Results

### 3.1 Isolation of marine microorganisms

From twenty samples collected (10 samples from sediment and 10 samples from seawater) from 10 different sites along Tehama western coast of Yemen, 65 marine actinomycetes isolates were isolated (42 isolated from sediment & 23 isolates from seawater). Only 25 isolates were subjected for investigation, identification and antimicrobial screening in this study. Identification of the 25 strains by morphological, microscopical, and cultural characteristics revealed that 14 isolates (56%) were species of the genus *Streptomyces* including the microbial strain 1S1 (Fig. 2), 7 of isolates (28%) were assigned to the genus *Actinoplanes*, 1 isolate was identified as *Nocardiopsis* species (4%), and the remaining were unknown (Data are not shown).

### 3.2 Antimicrobial activity

The results are presented in Table 1. Thirteen actinomycete isolates (52%) out of the 25 isolated actinomycetes demonstrated a positive antimicrobial activity against the selected human pathogens (Table 1). The isolate samples 1S1, 1S4, 1S5 and 1S8 showed the greatest antimicrobial activity against almost tested pathogens with inhibition zones between 10 and 45 mm. The marine actinomycete isolate (1S1) was therefore chosen for further investigation.

**Table 1:** The antimicrobial activity (inhibition zones in mm) of most active marine actinomycete isolates from Yemeni coast against six pathogenic microbes.

Sample code	*Staphylococcus aureus* ATCC6538	*Escherichia coli* MTCC739	*Bacillus subitlis* ATCC6633	*Pseudomonas aeruginosa* MTCC2453	*Candida albicans* ATCC2091	local *Streptococcus pneumoniae*
7 W1	30	–	40	–	–	9
7 W2	29	–	35	–	–	–
7 W3	23	8	20	–	–	–
7 W6	20	9	28	9	–	9
1S1	40	20	45	18	–	25
1S4	35	9	40	18	21	20
1S5	35	20	45	–	20	15
1S8	35	20	40	17	19	17
13 W2	22	–	29	–	–	30
13 W4	–	20	25	–	–	22
4S1	35	20	45	–	–	–
6S1	26	15	35	12	15	30
3 W2	35	–	40	–	–	–

### 3.3 Identification of the isolate 1S1

The most promising isolate 1S1 that has a wider zone of inhibition, was chosen for further research. The isolate 1S1 was identified as *Streptomyces* genus based on mycelium formation and spiral chain development (Fig. 2 a-e). The isolate 1S1 is Gram positive strain which produced pale white colonies on SCA plates. The growth of the isolate on different types of media is shown in Table 2. Moreover, the additional characterization based on biochemical characteristics is demonstrated in Table 3.

**Table 2:** Growth of the marine isolate 1S1 on different media.

No. of isolates	Medium	Growth	Aerial mycelium	Substrate mycelium	Diffusible pigment
1S1	Starch casein agar	+++	White	White	–
	Starch nitrate agar	++	white	white	–
	Inorganic salt starch agar	++	White	Page	–
	Nutrient agar	+	Creamy	Creamy	–
	Sabouraud dextrose agar	+	Creamy	Creamy	–
	Potato dextrose agar	++	White	Reddish	Reddish
	Glycerol asparagine agar	++	White	Page	–
	Bennett’s agar	+	White	Light gray	–
	Tyrosine agar	+	White	Pale yellow	–
	Czapek Dox agar	+	White	White	–

+++: very good, ++: good, +: Scanty, -: no

**Table 3:** Morphological and Biochemical characteristics of the marine isolate 1S1.

Test character	Results	
Morphological characters		
Colony shape	Regular	
Colony edge	Entire	
Colony consistency	Dry-ash	
Colony elevation	Concave	
Spore grouping	Chain	
Spore color	Gray	
Spore chain	Hook	
Arial mycelium	White to gray	
Substrate mycelium	white	
Diffusible pigment	-	
Production of melanin pigment	Tyrosine agar	Tryptone-yeast extract broth
	-	-

	Growth under different physiological conditions										
	pH			NaCl in %			Temperature in °C				
	3	7.7	10	0	5	10	20	28	37	45	60
	+	+	-	+	+	+	+	+	+	+	-
Utilization of different carbon sources											

starch	+										
maltose	+++										
mannose	++										
lactose	-										
xylose	+										
galactose	++										
fructose	+										
glucose	-										
sucrose	+										
Sodium acetate	-										
glycerol	+										
Utilization of different nitrogen source											

L- alanine	++										
DL-Phenylalanine	+										
L- Asparagine	+										
L-Tyrosine	+										
L- Arginine	+										
L-Cystine	-										
NH_4_NO_3_	-										
(NH_4_)_2_SO_4_	+										
KNO_3_	+										
NaNO_3_	+										
Enzyme production											

Amylase	+++										
Gelatinase	-										
Chitinase	-										
Catalase	-										
Pectinase	+										
Arginase	-										
Licithinase	+										
Urease	+										
Lipase	+										
Cellulase	-										
Caseinase	-										

+++: very good growth, utilization or production;

++: good growth utilization or production;

+: Scanty growth, utilization or production;

-: no growth, utilization or production.

**Fig. 3:**
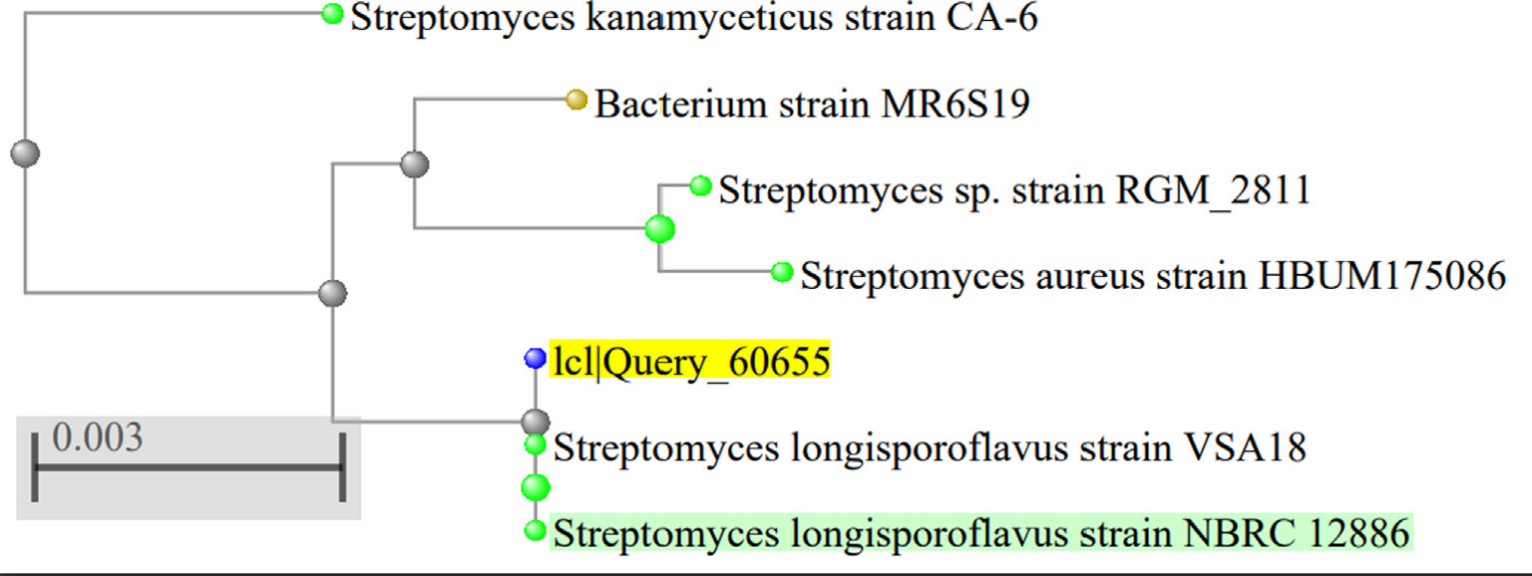
Phylogenetic tree of the actinomycete isolate (1S1) using 16S rRNA gene sequences. The similarity scores obtained from National Center for Biotechnology Information (NCBI) (https://blast.ncbi.nlm.nih.gov) confirm that the bacterial isolate is a *Streptomyces longisporoflavus*.

The 16S rRNA sequencing was carried out to identify the actinomycete strain 1S1. Fig. 3 shows the results of identification through PCR sequencing, and identifying the sequences by using the BLAST tool at NCBI. The results demonstrated the identification of the strain 1S1 as *Streptomyces longisporoflavus*.

### 3.4 Ethyl acetate extract analysis by GC–MS

Since the marine actinomycete strain 1S1 showed the greatest antimicrobial activity, it was selected for further chemical investigation. The fermentation broth of this bacterial strain was extracted with ethyl acetate and subjected to GC–MS analysis. The chemical composition of the investigated ethyl acetate extract, retention times and percentages are given in Table 4, where the identified components are listed in order of their elution on the Elite-5MS column. The GC–MS investigation led to the identification of 27 constituents. Comparing the mass spectra of the peaks with those in the NIST 11 MS data library helped to identify them. Overall, aliphatic acids (fatty acids) were determined as the major group, among which hexadecanoic acid (9.1%), 9-octadecenoic acid (7.8%) and 9,12,15-octadecatrienoic acid (7.5%) were found to be the main constituents (Table 4).

**Table 4:** Chemical composition of the ethyl acetate extract determined by GC-MS.

No.	Compound name	RT	Area	Area %
1	Hexanal	3.42	98,308	2.580
2	Butanoic acid	3.76	18,931	0.500
3	Nonanal	5.58	143,430	3.760
4	n-(4-Hydroxy-5-methylhexyl)benzene	5.98	57,446	1.510
5	Di-isobutyl succinate	6.33	37,592	0.990
6	Benzaldehyd	6.45	43,862	1.150
7	n-Ethyl-n-[(1-methylethoxy)methyl]-ethanamine	6.55	170,078	4.460
8	1,2,3-Propanetriol	7.27	285,666	7.500
9	Decanoic acid	7.48	52,216	1.370
10	2-Propenoic acid	7.83	18,399	0.480
11	Dodecanoic acid	8.81	146,898	3.860
12	3-Tetradecene	9.02	16,303	0.430
13	1-Undecanol	9.49	8624	0.230
14	Tagetonol	9.61	11,223	0.290
15	1,2-Benzenedicarboxylic acid	10.40	164,820	4.330
16	1-Heptadecanol	10.47	23,319	0.610
17	Methyl ester of heptadecanoic acid	10.65	39,977	1.050
18	Tridecanoic acid	10.80	170,049	4.460
19	Tetradecanoic acid	11.14	249,923	6.560
20	e-9-Tetradecenoic acid	11.52	171,085	4.490
21	Decanedioic acid	11.61	162,595	4.270
22	Dibutyl ester of1,2,3-propanetricarboxylic acid	11.91	673,091	7.660
23	5-Chlorocavernicoline	12.00	160,462	4.210
24	Erucylamide	12.14	886,056	3.250
25	9-Octadecenoic acid	12.21	297,092	7.810
26	Hexadecanoic acid	12.42	346,608	9.140
27	9,12,15-Octadecatrienoic acid	13.30	257,552	7.530

RT: retention time

### 3.5 Isolation and identification of the major compounds from the ethyl acetate extract

The extract was subjected to chromatographic separation techniques e.g., thin layer chromatography (TLC) and open column chromatography (CC) in order to isolate the main constituents. Ethyl acetate extract was chromatographed on a silica gel column and the collected fractions were pooled together depending on their TLC behavior to give six main fractions (scheme 1).

### 3.6 Characterization of compound 1

**Compound 1** has been acquired in the form of a white amorphous powder that dissolved readily in chloroform. The IR spectrum demonstrated the presence of strong bands at 1750 and 1165 cm^−1^ indicating an ester function (ester, C = O, C-O) and strong bands at 2910 and 2845 cm^−1^ indicating the presence of methylene groups. The EIMS spectrum of compound 1 displayed an [M + ] ion at *m*/*z* 722 consistent with the molecular formula C_45_H_86_O_6_. Several NMR-data including ^1^H, ^13^C, DEPT-135, HSQC, ^1^H–^1^H COSY and HMBC were achieved for the structure elucidation of this compound. The ^13^C NMR spectrum showed the presence of 45 carbon signals including signals at *δ* 173.49, 173,40 and 172.98 assigned to three ester carbonyl carbon atoms, a signal at *δ* 69.02 for methine carbon, two signals at *δ* 62.22 and 62.19 for methylene carbons indicating a triglyceride (ester of glycerol with three tetradecanoic acids). The ^13^C NMR-spectrum showed also 18 peaks between 29.10 and 29.95 ppm suggesting the presence of many CH_2_ C-atoms. That was assured by the DEPT 135˚ and HSQC spectra. In addition, a corresponding peak at 1.24–1.32 ppm in the ^1^H NMR showed that compound **1** represents probably a fatty acid ester. All the other signals at *δ* 22.70 to 36.88 were caused by CH_2_-groups C-atoms in a long chain. In addition, three methyl carbons at δC 14.26, 14.43 and 14.40 were indicated for three terminal methyl groups of three tetradecanoic acids.

**Fig. 4:**
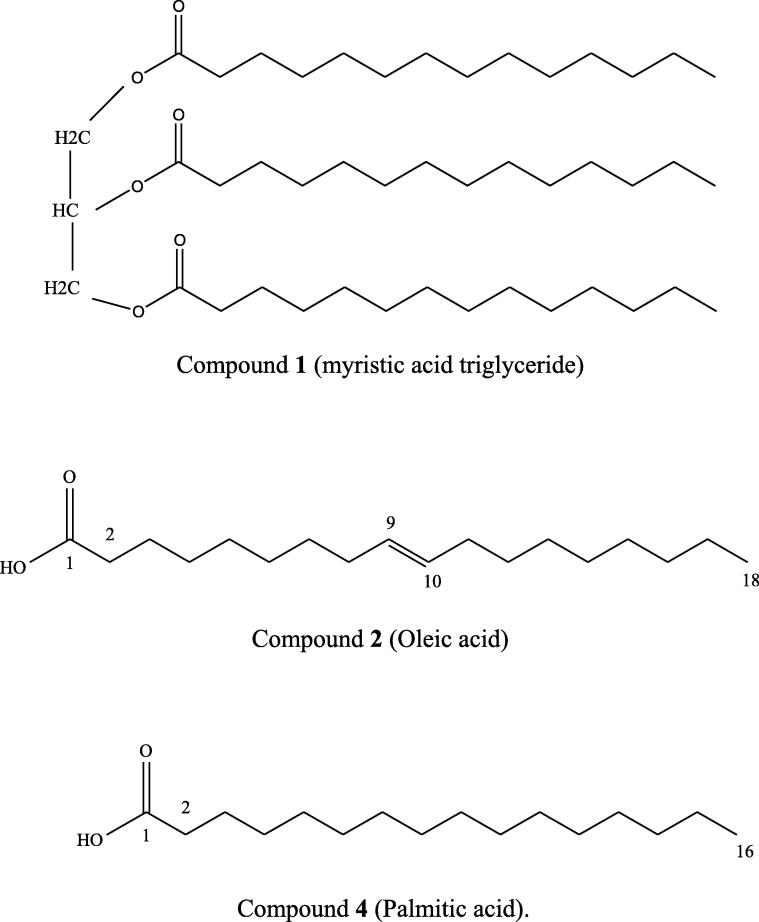
The chemical structures of the isolated compounds.

The ^1^H NMR spectrum of the compound **1** showed characteristic signals at *δ* 0.87, 0.88 and 0.89 as three triplets of three protons as an evidence of terminal CH_3_-groups, at *δ*1.29 as a broad singlet for a long chain of CH_2_-protons, at *δ* 2.88, 2.97 and 3.11 for CH_2_-groups α to carbonyl group, two signals at *δ* 4.12 and 4.15 with two double protons for CH_2_-groups and a signal at *δ* 4.28 for a methine group. The HSQC spectrum showed that the protons at *δ* 4.12 and 4.15 are attached on the methylene carbons at *δ* 62.22 and 62.19 and the proton at *δ* 4.28 was attached on methine carbon at *δ* 69.02. Relying on the NMR data and matching with literature data (Frank et al., 1971, Magritek NMR-resources, spectra-library., 2018), compound 1 was found to be a triacylglyceride of myristic acid (trimyristin) (Fig. 4).

### 3.7 Characterization of compound 2

**Compound 2** (3 mg) was obtained as brownish yellow oily substance. A number of important functional groups were visible in the IR spectrum. The IR spectrum revealed a weak band at 3502 cm^−1^ indicating the presence of OH-group, strong bands at 2920 and 2845 cm^−1^, suggesting the presence of methylene groups as well as a strong band at 1712 cm^−1^ showing the existence of a CO-group. The EIMS spectrum of compound 2 showed an [M + ] ion at *m*/*z* 282 proportionate with the molecular formula C_18_H_34_O_2_. The ^1^H NMR spectrum of compound **2** demonstrated one triplet signal of three protons at *δ* 0.87 indicating the presence of a terminal CH_3_-group. A broad singlet of twenty protons was displayed at *δ*1.29 for a long chain of CH_2_-protons. One multiplet of four protons at *δ* 2.01 was accounted for two CH_2_-groups α to C = C group (neighbors of the olefinic protons). The ^1^H NMR spectrum showed also one multiplet of two protons and one triplet of two protons at *δ* 1.63 and 2.22 assigned for CH_2_-groups β and α to CO-group respectively. Moreover, a triplet signal of two protons at *δ* 5.33 was attributed to olefinic protons. The ^13^C NMR spectrum showed the presence of 18 carbon signals including two signals in the unsaturated carbon region, at *δ* 130.02 and *δ* 130.06 for methine carbons. The signal at *δ* 175.82 appeared to be a carbonyl carbon atom of a carboxylic acid moiety. Additionally, one methyl carbon at *δ* 14.26 was indicated for the terminal methyl group. All the other signals at *δ* 22.82 to 36.67 were assigned to CH_2_-carbons in a long chain. Relying on the NMR data and matching with literature data (Malarvizhi et al., 2016), compound **2** was found to be oleic acid (Fig. 4).

### 3.8 Characterization of compound 4

**Compound4** (3.5 mg) was obtained as an oily white residue. The IR spectrum showed a weak band at 3500 cm^−1^ confirming the existence of a OH-group, strong bands at 2928 and 2841 cm^−1^ indicating the presence of methylene groups, and a strong band at 1715 cm^−1^ showing the presence of a CO-group. The EIMS spectrum of compound 4 displayed an [M + ] ion at *m*/*z* 256 consistent with the molecular formula C_16_H_32_O_2_. The ^1^H NMR spectrum of compound **4** demonstrated one triplet signal of three protons at *δ* 0.89 indicating the presence of a terminal CH_3_-group. A broad singlet of twenty-four protons was displayed at *δ*1.29 for a long chain of CH_2_-protons. The ^1^H NMR spectrum displayed also one multiplet of two protons and one triplet of two protons at *δ* 1.61 and 2.31 respectively, assigned for CH_2_-groups β and α to CO-group. The ^13^C NMR spectrum showed the presence of 16 carbon signals including a signal at *δ* 174.92 assigned to the a carbonyl C-atom of a carboxylic acid moiety. All the other signals at *δ* 22.70 to 36.88 were assigned to CH_2_-carbons in a long chain. In addition, one methyl carbon at *δ* 14.13 was indicated for the terminal methyl group. Relying on the NMR data and matching with literature data (Di Pietro et al., 2020, Magritek NMR-resources, spectra-library., 2018), compound 4 was found to be palmitic acid (C_16_H_32_O_2_) (Fig. 4).

## 4 Discussion

Antibiotic-resistant pathogenic microbes, infectious diseases, and new threats to human health and the economy have all increased in recent years. The insufficiency of terrestrial natural resources to solve these concerns has encouraged researchers to look for novel treatments in hitherto unexplored habitats. Marine environments including the Red Sea have become surprisingly rich sources of new biologically active substances, including antibacterial, anti-fungal, anti-inflammatory and anti-cancer agents (Nadeem et al., 2015). This study was carried out due to the scarcity of studies demonstrating the microbial richness of the Red Sea ecosystem in Yemen in comparison to the northern Red Sea. Twenty-five samples were collected from sediment and sea water from 10 different sites along Tehama western coast of Yemen. Fourteen of the isolates (56%) had distinct morphological, microscopic, and cultural traits indicating they belong to the genus *Streptomyces*. Actinomycetes in general are assumed to be the most economic and biotechnologically important prokaryotes producing many secondary and biologically relevant metabolites. According to Jakubiec-Krzesniak (2018) and co-authors, actinobacteria are among the most prolific natural makers of bioactive compounds, and they continue to be a significant source of novel secondary metabolites for medication development (Jakubiec-Krzesniak et al., 2018). In their review, they reported on the bioactive antimicrobial secondary metabolites of actinobacteria that have been discovered between 2011 and 2018. They focused in their research on the chemical structures, biological activity, and origin of these novel antibacterial, antifungal, and antiviral compounds, as well as their pharmacological properties. In terms of antibacterial activity, compounds like arenimycin C, chromopeptide lactone RSP 01, kocurin, macrolactins A1 and B1, chaxamycin D, and anthracimycin were considered to be the most effective chemicals (Jakubiec-Krzesniak et al., 2018). *Streptomyces* is an industrially important class of species, among these actinomycetes, which have been widely investigated for a broad range of bioactive constituents (Berdy, 2005, Dholakiya et al., 2017). The results showed the growth rate, macroscopic features (aerial mycelium and substrate mycelium), and diffusible pigment production of the *Streptomyces* sp. 1S1 depend on the growth medium used for cultivation. In addition to the above, the hook spore chain is considered as one of the most important microscopic features of this isolate. In general, the microscopic, macroscopic, and biochemical characteristics obtained in this study are largely compatible with features of *Streptomyces* strains which were documented in Bergey's Manual of Systematics of Archaea and Bacteria.

Thirteen out of the twenty-five samples showed remarkable antimicrobial activity. Comparison of 16S rRNA gene sequences showed that most antimicrobial active strain 1S1 is a member of the genus *Streptomyces*, showing highest similarity with *Streptomyces longisporoflavus*. In line with observations in studies such as those by Abdelmohsen et al., 2014, ElAhwany et al., 2015, our results confirm that marine bacteria and actinomycetes from the Red Sea exhibit antimicrobial activity. Researchers have found that 35% of the actinomycetes isolated from the Red Sea sponges demonstrate antimicrobial activity, as reported by Abdelmohsen et al. (2014). Similarly, 80% of the tested strains of actinomycetes isolated from the Red Sea corals have antimicrobial activity, as demonstrated by El-Ahwany et al. (2015). Additionally, our findings are in agreement with several recently published reports indicating that many isolated strains belonging to the genus *Streptomyces* possess interesting antimicrobial activity against Gram-positive and Gram-negative bacterial pathogens (Kumar et al., 2014, Dholakiya et al., 2017, Siddharth and Vittal, 2019, Shao et al., 2019). Few studies have been published on *S. longisporoflavus* which was isolated and identified in the current work, demonstrating the separation of biologically active compounds such as the polyketide-polyether antibiotic, tetronasin (C_35_H_54_O_8_) (Linton et al., 1994) as well as alkaloid-derivatives e.g., staurosporine (C_28_H_26_N_4_O_3_) (Cai et al., 1996).

The partial fractionation and purification of the ethyl acetate extract of *Streptomyces* sp. 1S1 performed using various chromatographic techniques, e.g., open column chromatography (CC), thin layer chromatography (TLC) and gas chromatography (GC), lead to the isolation of three compounds, which were identified as myristic acid triglyceride (trimyristin), 9-octadecenoic acid (oleic acid), and hexadecanoic acid (palmitic acid) (Fig. 4). The structure elucidation was carried out by ^1^H and ^13^C and 2D-NMR analysis and by comparison with literature data published previously (Di Pietro et al., 2020, Lu et al., 2013, Sopelana et al., 2013, Nieva-Echevarría et al., 2015).

Triacylglycerols (TAGs) are widely seen in yeast, fungi, plants, and animals (but not in bacteria) as fatty acid storages. Nevertheless, TAG accumulation appears to be popular among actinomycetes, including *Mycobacterium*, *Streptomyces*, *Rhodococcus*, and *Nocardia* species (Alvarez and Steinbüchel, 2002, Murphy, 1990, Murphy, 2001). Bacterial TAGs usually serve as spare substances. Because of their extreme hydrophobicity, TAGs can be synthesized in large quantities without significantly altering cytoplasm osmolarity. TAGs may also be used to reduce bacterial cell equivalents if terminal electron acceptors are not supplied adequately as stated for PHA in aerobic bacteria (Alvarez and Steinbüchel, 2002). Other functions discussed include the control of membrane fluidity of the cell, removal of uncommon fatty acids from membrane lipids and minimization of equivalents. Bacterial TAGs may additionally serve a role in maintaining membrane lipid fatty acids composition to adapt its fluidity to its environment. TAGs may either be used as a fatty acid donor for phospholipid biosynthesis or as a recipient of exceptional fatty acids, that might disrupt membrane fluidity otherwise (Murphy, 1990). Furthermore, TAGs may also serve as carbon source for antibiotics biosynthesis via the transformation of acetyl-CoA or malonyl-CoA to their respective products as previously observed by Olukoshi and Packter (1994).

Oleic acid is one of many unsaturated fatty acids, while palmitic acid is one of several saturated fatty acids; both can be found in many plants and marine organisms including marine algae and bacteria. Many fatty acids are known to be antimicrobial (Russel, 1991). Oleic acid has been found to be fungicidal when tested against a wide variety of mold and yeast species. There was 8-hour delay in fungal spore germination at low concentrations, which was discovered after running an experiment (Sheba et al., 1999). The fatty acids known to have antibacterial and antifungal properties include dodecanoic acid, tetradecanoic acid, hexadecanoic acid, octadecanoic acid, and oleic acid (Seidel and Taylor, 2004, McGraw et al., 2002). In 2012, Sandoval-Montemayor and co-authors reported that the hexane extract of the fruit peels of *Citrus aurantiifolia* showed activity against *Mycobacterium tuberculosis* H37Rv. The active extract was fractionated by column chromatography, yielding 6 major compounds including palmitic acid which was one of the most active ones.

Thus, it is anticipated that the fatty acids identified in this work, particularly 9-octadecenoic acid (oleic acid) and hexadecanoic acid (palmitic acid), may also be responsible for the antimicrobial effect of the *Streptomyces* sp. 1S1 ethyl acetate extract.

## 5 Conclusion

New bioactive natural products can be discovered by studying the marine actinomycetes of the Red Sea. Such natural products can particularly be found in the genus *Streptomyces*. In the current work, twenty-five bacterial species were isolated from twenty samples from the seashore sediments and water of west Yemen coast and evaluated for their antimicrobial action. Our findings showed that the most antimicrobial active strain was *Streptomyces* sp*.*. Analysis of the crude extract by GC–MS indicated the presence of certain acyl-fatty compounds. Three compounds were isolated and identified as myristic acid triglyceride, 9-octadecenoic acid, and hexadecanoic acid. In this study, the functionality of the Red Sea bacteria as a source of bioactive, biotechnologically, and pharmaceutically significant compounds was underlined.

## Funding

This research work was funded by Researchers Supporting Project number (RSP-2021/119), King Saud University, Riyadh, Saudi Arabia

## Declaration of Competing Interest

The authors declare that they have no known competing financial interests or personal relationships that could have appeared to influence the work reported in this paper
